# Linkage mapping of *Barley yellow dwarf virus* resistance in connected populations of maize

**DOI:** 10.1186/s12870-015-0420-x

**Published:** 2015-02-03

**Authors:** Frederike Horn, Antje Habekuß, Benjamin Stich

**Affiliations:** Max Planck Institute for Plant Breeding Research, Carl-von-Linné Weg, Cologne, 50829 Germany; Julius Kühn Institute, Erwin-Baur-Straße 27, Quedlinburg, 06484 Germany

**Keywords:** QTL analysis, BYDV, Connected populations, *Zea mays* L.

## Abstract

**Background:**

With increasing winter temperatures, *Barley yellow dwarf virus* (BYDV) is expected to become an increasing problem in maize cultivation in Germany. Earlier studies revealed that BYDV has a negative impact on maize performance. Molecular markers would accelerate the development of BYDV resistant maize. Therefore, the objectives of this study were (i) the identification of quantitative trait loci (QTL) for BYDV resistance in five connected segregating maize populations in a field experiment and (ii) their comparison with the QTL detected under greenhouse conditions.

**Results:**

In linkage analyses of the traits virus extinction, infection rate, and the symptom red edges, a highly associated major QTL was identified on chromosome 10. This QTL explained 45% of the phenotypic variance for the traits virus extinction and infection rate and 30% for the symptom red edges.

**Conclusion:**

We could show that BYDV resistance traits are oligogenically inherited. The QTL on chromosome 10 could be observed in the connected linkage analyses and in the single population analyses. Furthermore, this QTL could also be confirmed in the greenhouse experiment. Our results let suggest that this QTL is involved in multiple virus resistance and the markers are promising for marker assisted selection.

**Electronic supplementary material:**

The online version of this article (doi:10.1186/s12870-015-0420-x) contains supplementary material, which is available to authorized users.

## Background

Barley yellow dwarf (BYD) is one of the economically most important virus diseases in small grain cereals. *Zea mays* L. plays an important role as a summer host for the aphid-transmitted *Barley yellow dwarf virus* (BYDV) [1]. With increasing winter temperatures due to climate change, aphids overwinter anholocyclic. This leads to an earlier development of aphid populations with a higher number of aphids in spring. Consequently, the infestation pressure on maize by viruliferious aphids is increased [1]. Furthermore, aphids infect plants in early developmental stages [2], in which maize reacts with strong growth reduction on BYDV infection because of an incomplete organ development [1].

In earlier studies, a reduction of plant height, ear height, fresh yield, and grain yield as well as an earlier flowering was observed in BYDV inoculated maize compared to non-inoculated maize plants [3-5]. The control of the virus directly is not possible. The aphids could be controlled by application of an insecticide. However, in Germany the application of insecticides against aphids in maize is not allowed. Thus, genetic resistance is the only alternative. Furthermore, with resistant maize cultivars, the BYDV transmission cycle can be broken and with this, the situation in cereals could be improved as well.

Characteristic symptoms of BYDV infected maize are red bands at the edge of the leaves. These symptoms correlate positively with the virus extinction measured by double-antibody sandwich enzyme-linked immunosorbent assay (DAS-ELISA) and is a fair indicator for susceptibility [6]. Nevertheless, tolerant genotypes which do not show any symptoms but high virus extinction exist [6-8]. To identify resistant genotypes, it is therefore necessary to measure the virus extinction by DAS-ELISA. The evaluation of maize genotypes for their BYDV resistance by aphid inoculation and DAS-ELISA analyses, however, is very labor and cost intensive and therefore its integration in practical breeding programs is not reasonable. Therefore, the identification of genome regions which are linked to BYDV resistance and the application of this knowledge in marker assisted selection (MAS) programs of maize would allow a faster progress in breeding of BYDV resistant maize.

In previous studies, resistance loci have been identified for various viruses in maize. On chromosome 10, three minor quantitative trait loci (QTL) were identified for *Sugarcane mosaic virus* (SCMV) resistances [9,10]. Zambrano et al., 2014 [11] identified a resistance locus on chromosome 1 for *Maize mosaic virus* (MMV) and two resistance loci on chromosome 10 for *Wheat strike mosaic virus* (WSMV) and *Maize dwarf mosaic virus* (MDMV). For WSMV, also McMullen and Simcox, 1995 [12] as well as Jones et al., 2011 [13] identified a resistance locus on chromosome 10. On chromosome 10, further resistance loci were found for *Maize chlorotic dwarf virus* (MCDV) [14]. But, to the best of our knowledge, no results from QTL mapping of BYDV resistance in maize are available. However, in a genome wide association study (GWAS) Horn et al., 2014 [15] identified single nucleotide polymorphisms (SNPs) on chromosome 4 and 10 in a diverse germplasm set, representing the world-wide maize diversity [16], which explained a high proportion of phenotypic variation for traits related to BYDV resistance.

The disadvantage of GWAS is that genes contributing to phenotypic variation which show a low allele frequency can remain undetected. Due to a balanced allele frequency in segregating populations, classical linkage mapping has the advantage of higher QTL detection power compared to GWAS [17]. In the GWAS of Horn et al., 2014 [15], population structure was taken into account to avoid the detection of spurious associations [16]. However, with this correction it is possible that QTL effects are absorbed in population effects and stay undetected in a GWAS [18]. Therefore, in this study we used linkage mapping to be able to detect additional QTL for BYDV resistance and furthermore, if possible, validate the genome regions identified previously by GWAS.

In this study, five connected segregating populations were used to map QTL for BYDV resistance. In connected QTL mapping populations, the probability to find alleles of interest is higher because more than two alleles can be considered in multiple genetic backgrounds in contrast to a single biparental population [19] and therefore this approach increases the probability that a QTL will be polymorphic in at least one population [20]. The objectives of this study were (i) the identification of QTL for BYDV resistance in five connected segregating maize populations in a field experiment and (ii) their comparison with the QTL detected under greenhouse conditions.

## Material and methods

### Phenotypic evaluation of five connected segregating populations

Five connected segregating maize populations with a total of 443 entries [A: n=85 (F _2:3_), B: n=83 (F _3:4_), C: n=77 (F _4:5_), D: n=92 (F _4:5_), E: n=106 (F _4:5_)] derived from biparental crosses of five inbred lines were examined in our study (Table 1).

**Table 1:** **Population means and adjusted means of the parental inbreds for the traits red edges (RE), virus extinction (EX), and infection rate (IR) from the field experiment, and for the traits EX and IR from the greenhouse experiment**

	**Field**				**Greenhouse**	
**Entry type**	**RE**	**EX**	**IR [%]**		**EX**	**IR [%]**
Segregating populations						
Population A (P092 x FAP1360A)	1.42	0.66	54.57		0.68	54.04
Population B (P092 x Ky226)	1.50	0.49	41.21		0.87	80.46
Population C (Ky226 x FAP1360A)	1.17	0.21	13.57		0.50	37.70
Population D (D408 x W64A)	3.69	0.76	66.38		0.60	53.45
Population E (D408 x P092)	1.37	0.66	55.50		0.61	53.44
Parental inbreds						
Ky226 (resistant)	1.45	0.30	19.88		0.39	27.88
W64A (susceptible)	7.15	0.83	80.66		0.77	82.72
FAP1360A (resistant)	0.92	0.26	18.28		0.76	67.32
P092 (tolerant)	0.89	1.02	80.79		1.15	85.40
D408 (tolerant)	1.92	0.76	60.44		0.46	35.62

The field experiments with these populations were carried out at Borken and Wadersloh (both Germany) in 2011 and 2012. Each population was planted separately in a single trial and the experimental design of each trial was an *α* lattice design, where the five parental inbreds served as repeated checks.

Two weeks after sowing, the plants were inoculated with BYDV. For the transmission of the virus, aphids of the genus *Rhopalosiphum padi* were raised for three weeks at 20°C on plants of the *Triticum aestivum* cultivar “Tuareg” which were infected with the virus BYDV-PAV. For the inoculation, a piece of wheat leaves with viruliferious aphids was placed in the leaf axil of each maize plant. Afterwards, the plants were covered with fleece *Climatex* (17g/qm) to prevent escape of the aphids. One week after inoculation, the fleece was removed and the insecticide “Biscaya” (Bayer, 300 ml in 200 to 400 l water/ha) was applied in the field.

Six weeks after inoculation, the BYDV symptom red edges (RE) was scored on a scale from 1 to 9 (1 = no symptoms, 9 = highest symptom expression) in the field experiments. We collected leaf material from the sixth leaf of each plant per row and measured virus extinction (EX) by DAS-ELISA as described by Horn et al., 2013 [6]. The infection rate (IR) was calculated as the percentage of plants of one plot with EX ≥0.5 [15].

In order to evaluate the five segregating populations under artificial conditions, controlling for light, temperature, humidity, soil nutrients, and water application, they were grown in a greenhouse experiment with two replicates, each with 10 plants per genotype. Each population was planted separately in a single trial and the experimental design of each trial was an *α* lattice design, where the five parental inbreds served as repeated checks. Experimental conditions (light, temperature, soil) were as described by Horn et al., 2014 [15] and in contrast to field experiment the insecticide “Lizetan Plus” (Bayer) was applied one week after inoculation. The evaluation of EX and IR in the greenhouse was assessed in the same way as described above for the field experiment.

### Statistical analyses

#### Phenotypic evaluation of five connected segregating populations

We used the mixed model (1) to analyse the data collected for the checks in the field experiments. As no obvious year effect was observed, we considered each year-location combination as one environment:

(1) $$\begin{array}{@{}rcl@{}} Y_{ijk} = \mu + R_{i} + T_{j} + C_{k} + e_{ijk}, \end{array} $$

where *Y*_*ijk*_ was the phenotypic observation for the *k*th check in the *j*th trial in the *i*th environment, and *μ* the general mean. In the analysis of the field experiment *R*_*i*_ was the effect of the *i*th environment, *T*_*j*_ the effect of the *j*th trial, *C*_*k*_ the effect of the *k*th check, and *e*_*ijk*_ the residual error. All effects, except *e*_*ijk*_ were regarded as fixed. The trial effect *T*_*j*_ was subtracted from the raw data of all entries of the corresponding trial to correct for the differences among the different trials. The adjusted data of all entries from the field experiment were then analyzed according to the following linear mixed model:

(2) $$\begin{array}{@{}rcl@{}} Y_{ijlmn} = \mu + R_{i} + D_{l}(TG)_{jm} + (RB)_{in} + e_{ijlmn}, \end{array} $$

where *Y*_*ijlmn*_ was the phenotypic observation for the *m*th entry in the *i*th environment in the *n*th incomplete block of the *j*th trial corrected for *T*_*j*_. *G*_*m*_ was the effect of the *m*th entry, *B*_*n*_ the effect of the *n*th block, and *e*_*ijlmn*_ the residual error. (*D*_1−5_) _*l*_ was a indicator variable with *D*_*l*_=04 for checks and *D*_*l*_=1−5 for the entry of the 1st - 5th trial which enabled the calculation of specific genotypic $\sigma ^{2}_{\textit {gj}}$ and error $\sigma ^{2}_{\textit {ej}}$ variances for the entries of the *j*th trial. *R*_*i*_ was regarded as fixed, whereas the *D*_*l*_(*TG*)_*jm*_ interactions and the (*RB*)_*in*_ interaction were regarded as random.

Formula (1) and (2) were also used for the analysis of the greenhouse experiment where *R*_*i*_ was the effect of the *i*th replicate.

Broad-sense heritability ${H^{2}_{j}}$ was calculated for each *j*th trial based on the formula

(3) $$\begin{array}{@{}rcl@{}} {H^{2}_{j}}= \frac{\sigma^{2}_{gj}}{\sigma^{2}_{gj} + \frac{\sigma^{2}_{ej}}{n}}, \end{array} $$

where n was the number of environments.

Broad-sense heritability on a plot basis $H^{*2}_{j}$ was calculated based on the formula

(4) $$\begin{array}{@{}rcl@{}} H^{*2}_{j}= \frac{\sigma^{2}_{gj}}{\sigma^{2}_{gj} + \sigma^{2}_{ej}}, \end{array} $$

For each entry, an adjusted entry mean was calculated as:

(5) $$\begin{array}{@{}rcl@{}} M_{m} = {\widehat{\mu}} + {\widehat{G_{m}}}, \end{array} $$

where $\widehat {\mu }$ was the estimate for the intercept and $\widehat {G_{m}}$ the estimate of the genetic effect of the *m*th entry calculated based on formula (2).

All mixed model analyses were performed with the software ASReml [21]. For each segregating population, the correlation coefficients among all pairs of traits were calculated. If not stated differently, all analyses were performed with the statistical software R [22].

#### Genotyping and consensus map construction

Plant material was collected from the leaves of each genotype and deoxyribonucleic acid (DNA) was isolated using *BioSprint 96*. The five parental inbreds were genotyped by the TraitGenetics GmbH (Gatersleben, Germany) with the MaizeSNP50 array [23]. Out of this set of 56.110 SNPs, we selected 163 SNPs which were equally distributed across the genome. Furthermore, we selected markers which were homozygote in the parental inbreds and were polymorphic in the highest number of segregating populations. These selected SNPs were genotyped for all five segregating populations with KASP marker technology by TraitGenetics GmbH.

A chi-square test was performed to test whether the SNPs deviate from the expected 1:1 ratio and SNPs which significantly (*α*=0.001) deviated from this ratio were excluded from further analyses [24]. For an improvement of the consensus map construction, further marker information of six connected segregating populations (Frey F, Stich B. Identfication of genome regions contributing to variation of heat tolerance in temperate maize by QTL mapping with multiple connected populations: Unpublished.), genotyped with the same marker set, were included. According to the position on the physical map, SNPs were assigned to their chromosomes and for each chromosome a consensus map was created using the software CarthaGène [25].

#### Linkage mapping

The linkage analyses with the software MCQTL [26] was based on the consensus map and the adjusted entry mean of each genotype. At first, we performed a single population analysis for each segregating population [19,20]. In the model we included additive and dominance effects because there are still heterozygous genotypes in our F _2:3_, F _3:4_, and F _4:5_ populations. To consider relationships between the populations due to shared parental inbreds, we furthermore performed a connected analysis [19,20] including also additive and dominance effects. For QTL detection, 0.01 quantile F thresholds were determined for each trait by 1,000 permutations. F thresholds for the cofactor selection were fixed at 90% of the F threshold values for QTL detection as proposed by the MCQTL software. The QTL detection was performed using an iterative composite interval mapping approach (iQTLm) [27]. SNP markers associated with the respective trait were selected as cofactors by forward regression considering a minimal distance of 10 cM between two selected cofactors [19].

To test, if the dominance effects of the populations were significantly different from 0, we calculated significance (*α*=0,05) a posteriori from a normal distribution using a two-sided test (personal communication, B. Mangin, August, 2014). The difference of the additive effects among pairs of alleles was tested a posteriori using a multicomparison t-test (Tukey) with *α*=0,05.

All genes between the physical position of the flanking markers from significant (*α*=0.01) QTL were extracted from the filtered gene set of the maize genome sequence ZmB73 _5b _FGS.

Using MCQTL, we performed tests among all pairs of marker loci to detect epistatic interactions with a model including additive, dominance, and epistatic effects and all identified QTL [28]. To make the allelic QTL (or cofactors) main effects estimable, we used the same constraints as in the QTL analysis for the single populations as well as for the connected analysis. For the detection of epistatic effects, 0.01 quantile F thresholds were determined by 1,000 permutation tests using the option “genowideforepistasy”.

## Results

**Figure 1:**
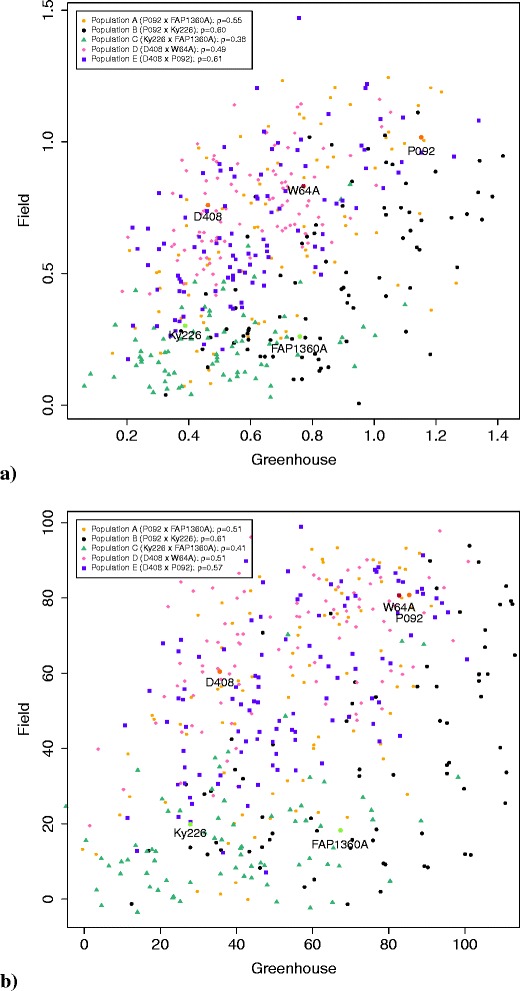
**Correlation of (a) the traits virus extinction (EX) and (b) infection rate (IR) between greenhouse and field experiment.** Each genotype is colored depending on the population it belongs to. Parental inbreds are lettered with their names and colored green (resistant), orange (tolerant) and red (susceptible). The legend shows the correlations of each population.

In the field experiments, the parental inbred P092 showed no symptoms (RE) but the highest value for EX and IR (Table 1). The strongest symptoms, however, were observed in the parental inbred W64A, which showed the second highest EX values. The lowest values for EX were observed for the parental inbreds Ky226 and FAP1360A. Population D showed the highest adjusted means for the trait RE and also the highest EX and IR compared to the other segregating populations. Population C showed the lowest values for RE, EX, and IR.The assessments made in the greenhouse and the field experiments correlated significantly (*α*=0.01) positive with *ρ*=0.43 for EX and *ρ*=0.44 for IR (Figure 1). For the single populations, the correlations varied for EX with *ρ* between 0.38 and 0.61 and for IR between 0.41 and 0.61. The lowest correlation was observed for population C for both traits.

Mean EX and IR values of the populations B and C were double as high in the greenhouse as in the field experiment (Table 1). The mean value of EX and IR were three times higher for the parental inbred FAP1360A in the greenhouse compared to the field experiment. On the other hand, the adjusted entry mean of both traits for the parental inbred D408 were double as high in the field experiment compared to the greenhouse experiment.

In the field experiment, we observed ${H^{2}_{j}}$ from 0.69 to 0.92 for the trait RE in the different segregating populations (Table 2). EX and IR showed high ${H^{2}_{j}}$ in all segregating populations (0.69-0.88). In the greenhouse experiment, the ${H^{2}_{j}}$ for EX (0.50-0.74) and IR (0.50-0.64) across the two replications was lower than the values observed in the field experiment for EX and IR.

**Table 2:** **Broad-sense heritabilities of the single populations for the traits traits red edges (RE), virus extinction (EX), and infection rate (IR) from the field experiment, and for the traits EX and IR from the greenhouse experiment**

	**Field**				**Greenhouse**	
**Entry type**	**RE**	**EX**	**IR**		**EX**	**IR**
Segregating populations						
Population A (P092 x FAP1360A)	0.76	0.87	0.85		0.74	0.62
Population B (P092 x Ky226)	0.84	0.86	0.88		0.68	0.63
Population C (Ky226 x FAP1360A)	0.73	0.82	0.85		0.67	0.56
Population D (D408 x W64A)	0.92	0.69	0.59		0.50	0.50
Population E (D408 x P092)	0.69	0.83	0.75		0.68	0.64

In the connected linkage analysis of the traits assessed in the field experiment, we identified a QTL with significant additive effects on the bottom of chromosome 10 at the position of 46.90 cM for the traits EX, IR, and RE. This QTL with significant additive effects explains a high proportion of the phenotypic variance for the traits EX (45%), IR (46%), and RE (30%) (Table 3). In the single population analyses, the genome positions on chromosome 10, which showed the maximum LOD score, varied among the examined populations. However, the confidence intervals (CI) of the QTL with significant additive effects on chromosome 10 for EX and IR overlapped with the CI of the connected analysis, except in population C (Table 4 and Table 5). Furthermore, in the single population analysis a QTL with significant additive and dominance effects was identified on chromosome 3 for EX in population D.

**Table 3:** **Chromosomic locations and confidence intervals (CI) of QTL with their flanking markers and number of genes within the CI from the connected analysis for the traits red edges (RE), virus extinction (EX), infection rate (IR) from the adjusted means of the field experiment, and for the traits EX and IR from the adjusted means of the greenhouse experiment**

**Trait**	**Chr**	**Pos [cM]**	**LOD**	**R** ^**2**^	**CI [cM]**		**Flanking**			**Physical**			**Number**
							**markers**			**interval [bp]**			**of genes**
Field													
EX	10	46.9	76.88	45	45-49		PUT-163a-60352819-2700	SYN15407		127005619	134855671		224
IR	10	46.9	80.26	46	45-51		PUT-163a-60352819-2700	SYN15407		127005619	134855671		224
RE	2	55.1	11.23	11	33-59		SYN29639	PZE-102117636		49294378	158105037		1173
	10	52.1	39.76	30	44-52		PUT-163a-60352819-2700	SYN15407		127005619	134855671		224
	Simultaneous fit			36									
Greenhouse													
EX	10	46.9	37.15	29	44-52		PUT-163a-60352819-2700	SYN15407		127005619	134855671		224
IR	9	24.2	9.72	10	17-37		PZA03759.2	PZE-109068137		84335878	112017256		433
	10	46.9	38.51	30	44-52		PUT-163a-60352819-2700	SYN15407		127005619	134855671		224
	Simultaneous fit			36

**Table 4:** **Chromosomic location and effects of QTL from the connected analysis for the BYDV resistance traits red edges (RE), extinction rate (EX), infection rate (IR) from the adjusted means of the field experiment, and for the traits EX and IR from the adjusted means of the greenhouse experiment**

				**Estimated additive allele effects**						**Estimated dominance allele effects**				
**Trait**	**Chr**	**Pos [cM]**		**Ky226**	**W64A**	**FAP1360A**	**P092**	**D408**		**Pop A**	**Pop B**	**Pop C**	**Pop D**	**Pop E**
Field														
EX	10	46.9		-0.09 ^*B*^	0.05 ^*A*^	-0.09 ^*B*^	0.18 ^*C*^	-0.06 ^*B*^		-0.01 ^*n*.*s*.^	-0.03 ^*n*.*s*.^	0.07 ^*n*.*s*.^	0.03 ^*n*.*s*.^	-0.04 ^*n*.*s*.^
IR[%]	10	46.9		-9.27 ^*B*^	7.19 ^*A*^	-8.01 ^*B*^	14.65 ^*A*^	-4.57 ^*B*^		0.59 ^*n*.*s*.^	-1.12 ^*n*.*s*.^	5.44 ^*n*.*s*.^	4.03 ^*n*.*s*.^	-4.16 ^*n*.*s*.^
RE	2	55.1		0.04 ^*B*^	-0.45 ^*A*^	-0.01 ^*AB*^	0.18 ^*B*^	0.23 ^*B*^		0.31 ^*n*.*s*.^	0.06 ^*n*.*s*.^	-0.03 ^*n*.*s*.^	-0.14 ^*n*.*s*.^	0.05 ^*n*.*s*.^
	10	52.1		-0.20 ^*BC*^	1.05 ^*A*^	-0.42 ^*B*^	-0.11 ^*C*^	-0.33 ^*BC*^		0.12 ^*n*.*s*.^	0.12 ^*n*.*s*.^	-0.08 ^*n*.*s*.^	0.10 ^*n*.*s*.^	-0.09 ^*n*.*s*.^
Greenhouse														
EX	10	46.9		-0.07 ^*B*^	0.11 ^*A*^	-0.08 ^*B*^	0.10 ^*A*^	-0.06 ^*B*^		0.01 ^*n*.*s*.^	-0.02 ^*n*.*s*.^	0.03 ^*n*.*s*.^	0.00 ^*n*.*s*.^	0.00 ^*n*.*s*.^
IR [%]	9	24.2		-7.42 ^*A*^	2.33 ^*ABC*^	-3.74 ^*AB*^	4.58 ^*C*^	4.24 ^*BC*^		-27.94 ^∗^	15.58 ^∗^	7.74 ^*n*.*s*.^	8.44 ^*n*.*s*.^	-6.56 ^*n*.*s*.^
	10	46.9		-6.49 ^*B*^	13.65 ^*A*^	-8.08 ^*B*^	8.75 ^*A*^	-7.84 ^*B*^		4.04 ^*n*.*s*.^	-3.02 ^*n*.*s*.^	0.67 ^*n*.*s*.^	-3.71 ^*n*.*s*.^	0.13 ^*n*.*s*.^

^*^, ^**^, ^***^, significant with significance level 0.05, 0.01 and 0.001, respectively.

^*ns*^, not significant.

^*A*^, ^*B*^, ^*C*^, populations with the same letter are not significantly (*α*= 0.05) different from each other.

**Table 5:** **Chromosomic locations and effects of QTL from the single population analysis for the traits red edges (RE), virus extinction (EX), infection rate (IR) from the adjusted means of the field experiment, and for the traits EX and IR from the adjusted means of the greenhouse experiment**

**Trait**	**Chr**	**Pos**	**CI**	**R** ^2^	**Estimated additive**	**Estimated**
					**allele effects**	**dominance allele**
					**parental inbred (1)**	**effects**
Population A (FAP1360A(1) x P092(2))						
Field						
EX	10	51.9	41-52	47	-0.12 ^∗^	0.02 ^*n*.*s*.^
IR [%]	10	51.9	41-52	46	-10.01 ^∗^	3.40 ^*n*.*s*.^
Greenhouse						
EX	10	51.9	39-52	31	-0.09 ^∗^	-0.06 ^*n*.*s*.^
IR [%]	10	52.1	39-52	31	-7.01 ^∗^	-3.78 ^*n*.*s*.^
Population B (Ky226(1) x P092(2))						
Field						
EX	10	51.9	50-52	68	-0.13 ^∗^	-0.01 ^*n*.*s*.^
IR [%]	10	52.1	50-52	71	-12.62 ^∗^	-1.18 ^*n*.*s*.^
Greenhouse						
EX	1	123.9	55-149	21	-0.05 ^∗^	0.14 ^∗^
	10	51.9	46-52	49	-0.10 ^∗^	-0.03 ^*n*.*s*.^
IR [%]	9	24.2	14-37	25	-6.99 ^∗^	14.55 ^∗^
	10	52.1	44-52	36	-8.53 ^∗^	-2.26 ^*n*.*s*.^
Population C (FAP1360A(1) x Ky226(2))						
Greenhouse						
EX	5	80.7	54-192	23	-0.18 ^∗^	-0.84 ^*n*.*s*.^
IR [%]	5	176.1	69-193	25	-11.03 ^∗^	11.09 ^*n*.*s*.^
Population D (W64A(1) x D408(2))						
Field						
EX	3	117.6	102-147	21	-0.05 ^∗^	-0.19 ^∗∗^
	10	44.8	33-52	30	0.06 ^∗^	0.04 ^*n*.*s*.^
IR [%]	10	44.8	34-52	30	6.06 ^∗^	4.19 ^*n*.*s*.^
RE	2	42.2	26-62	21	-0.37 ^∗^	-0.10 ^*n*.*s*.^
	10	52.1	39-52	47	0.69 ^∗^	0.12 ^*n*.*s*.^
Greenhouse						
EX	10	46.9	41-52	49	0.08 ^∗^	0.00 ^*n*.*s*.^
IR [%]	10	51.9	42-52	53	10.68 ^∗^	-2.91 ^*n*.*s*.^
Population E (P092(1) x D408(2))						
Field						
EX	10	39.8	37-47	48	0.13 ^∗^	-0.05 ^*n*.*s*.^
IR [%]	10	39.8	37-44	53	10.72 ^∗^	-5.22 ^*n*.*s*.^
Greenhouse						
EX	10	46.9	39-51	27	0.08 ^∗^	0.00 ^*n*.*s*.^
IR [%]	10	46.9	41-51	30	7.91 ^∗^	0.28 ^*n*.*s*.^

^*^, ^**^, ^***^, significant with significance level 0.05, 0.01 and 0.001, respectively.

^*ns*^, not significant.

A second QTL with significant additive effects for RE was identified on chromosome 2, explaining 11% of the phenotypic variance in the connected analysis. In the single population linkage analysis, this QTL CI with significant additive effects was only significant in population D.

In the greenhouse experiment, we observed significant QTL with significant additive effects for EX and IR which colocalized with the QTL on chromosome 10 identified in the field experiment in the connected analysis. Furthermore, in the single population analyses the CI of the QTL with significant additive effects for EX and IR overlapped with the CI of the connected analysis, except in population C. A QTL with significant additive and dominance effects was identified on chromosome 9 for IR in the connected analysis and in the single population analysis for population B. In the single population analysis, we identified in population C a QTL with significant additive effects for IR at 176.10 cM and a QTL with significant additive effects for EX at 80.70 cM on chromosome 5. For the latter QTL on chromosome 5 at 80.7 cM, we observed a significant (*α*=0.01) epistatic interaction with a genome position on chromosome 6 at 60.8 cM for the trait EX, explaining 21% of the phenotypic variance (Table 6).

**Table 6:** **Virus extinction of genotypes from population C from the greenhouse experiment and (the epistatic additive-additive effects) at the allele combination at the epistatic interacting genome positions**

	***Genotype at marker within***		
	***QTL on chromosome 5***		
**Allele (Effect)**	**GG (-0.16)**	**GA (-1.15)**	**AA (0.16)**
*Genotype at the epistatically interacting marker on chromosome 6*			
TT (-0.02)	0.44 (0.11)	0.53	0.45 (-0.11)
TC (0.24)	0.56	0.53	0.68
CC (0.02)	0.38 (-0.11)	0.39	0.71 (0.11)

## Discussion

### Comparison of field and greenhouse experiments for the assessment of BYDV resistance

The high ${H^{2}_{j}}$ values observed for the field and greenhouse experiments indicated that the traits are under a strong genotypic control and, thus, an improvement of the genotypes regarding these traits is possible by breeding. The ${H^{2}_{j}}$ values observed for the traits EX and IR under greenhouse conditions were lower than these observed under field conditions. The reason could be that in the calculation of ${H^{2}_{j}}$ in the field experiments n =4 due to the four environments whereas in the greenhouse n =2 due to the two replicates. Therefore, ${H^{2}_{j}}$ on a plot basis $\left (H^{*2}_{j}\right)$ was calculated to be able to compare the $H^{*2}_{j}$ values directly [29]. The $H^{*2}_{j}$ was more similar between the field and greenhouse experiments but still slightly higher for EX in the field (0.36-0.62) compared to $\left (H^{*2}_{j}\right)$ for EX in the greenhouse (0.33-0.58). This finding can be explained by the higher number of plants per plot in the field (15 plants) compared to the greenhouse (10 plants). Furthermore, $\sigma ^{2}_{\textit {gj}}$ and $\sigma ^{2}_{\textit {ej}}$ were calculated across four environments in the field compared to two replicates in the greenhouse. This could be the reason why $\sigma ^{2}_{\textit {ej}}$ becomes smaller in the field leading to slightly higher $H^{*2}_{j}$ in the field experiments.

We observed a significant (*α*=0.01) positive correlation between the BYDV resistance measured by EX and IR in the field and greenhouse experiments (Figure 1). This shows that our plant material reacts similarly to BYDV infection in the greenhouse and in the field. Nevertheless, the correlation was not tight with a correlation coefficient of 0.43 and 0.44. The difference between field and greenhouse results can be explained by the differences in growing conditions such as light, temperature, humidity, soil nutrients, water application and plant density. The different reaction of the genotypes to BYDV inoculation, resulting from different environmental conditions, are called genotype-environment interactions. Especially in population B and C the genotype-environment interaction led to higher EX and IR values in the greenhouse compared to the field experiments (Table 1 and Figure 1).

For EX and IR, the correlation between the field and greenhouse experiments was the lowest in population C compared to the other populations because the EX and IR values showed a much higher EX and IR values in the greenhouse experiment compared to the field experiments. Furthermore, we observed that one of the parental inbreds of population C, FAP1360A, which was resistant in the field experiment, showed high EX and IR values in the greenhouse experiment. In contrast, the parental inbred D408 showed lower EX and IR values in the greenhouse compared to the field experiment. In earlier studies, Grüntzig and Fuchs, 2000 [30] described the parental inbred D408 as resistant, which is in accordance to the greenhouse results. The reason for this finding could be that D408 reacts differently to BYDV infection, depending on the environmental conditions. However, this requires further research.

### Consensus map

**Figure 2:**
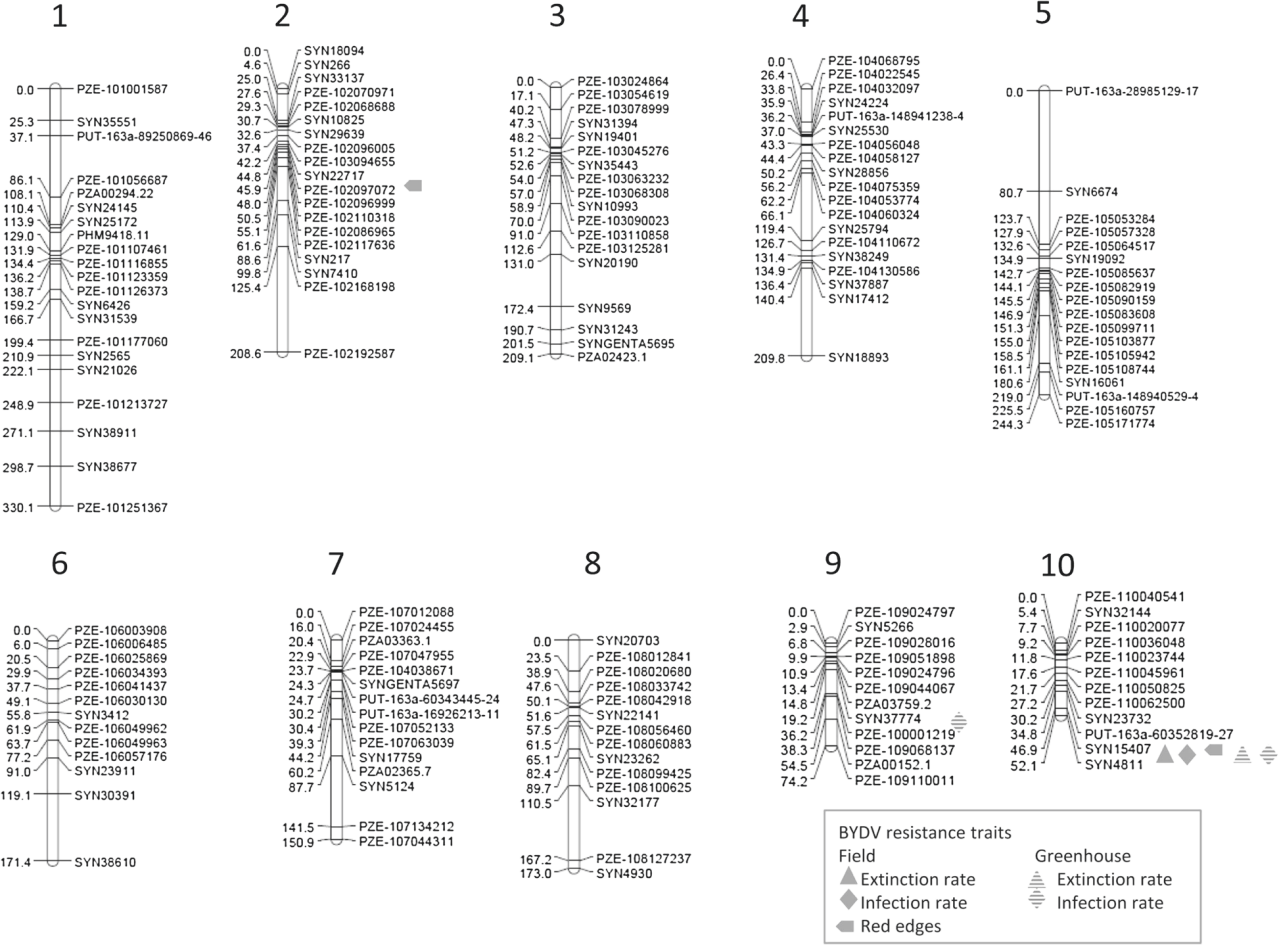
**Projection of the QTL identified in this study on the genetic consensus map.** QTL are illustrated by symbols for the traits red edges (RE), virus extinction (EX), and infection rate (IR) from the field experiment, and for the traits EX and IR, from the the greenhouse experiment.

The advantage of a consensus map created with a higher number of populations compared to a linkage map created with a single biparental population is the availability of more genotypic information, which improves the construction of the genetic map. Furthermore, more alleles can be taken into account simultaneously and therefore, the probability that at least one population is polymorphic at a given marker locus is higher. In our study, the consensus map was constructed across all five segregating populations examined plus six additional segregating populations from a companion study of Frey and Stich, unpublished (Frey F, Stich B. Identfication of genome regions contributing to variation of heat tolerance in temperate maize by QTL mapping with multiple connected populations:), which were genotyped with the same marker set. With this we reached an even higher marker information and a better coverage of markers over the genome for the consensus map than only across five populations (Figure 2).

**Figure 3:**
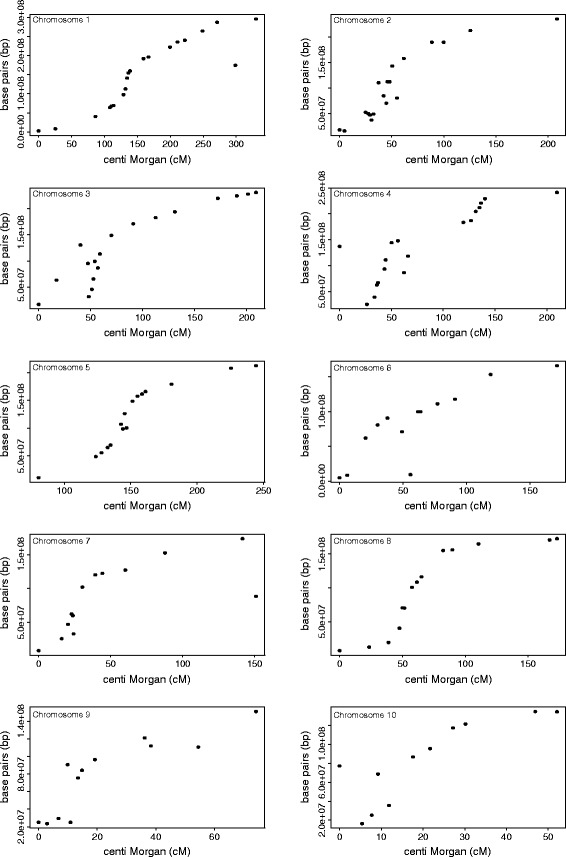
**Plot of the physical map position [bp] of the markers versus their genetic position [cM] on the consensus map.** The dots represent the markers used in our study on each of the 10 maize chromosomes.

The plot of genetic positions vs. the physical positions of the markers showed a sigmoid curve (Figure 3). The same pattern was observed by Payseur and Nachman, 2000 [31] and occurs because the recombination rate is lower at centromeres. However, we observed that the genetic map calculation is reasonable because the order of markers on the genetic map is consistent with the order of the physical map positions.

### QTL for BYDV resistance

**Figure 4:**
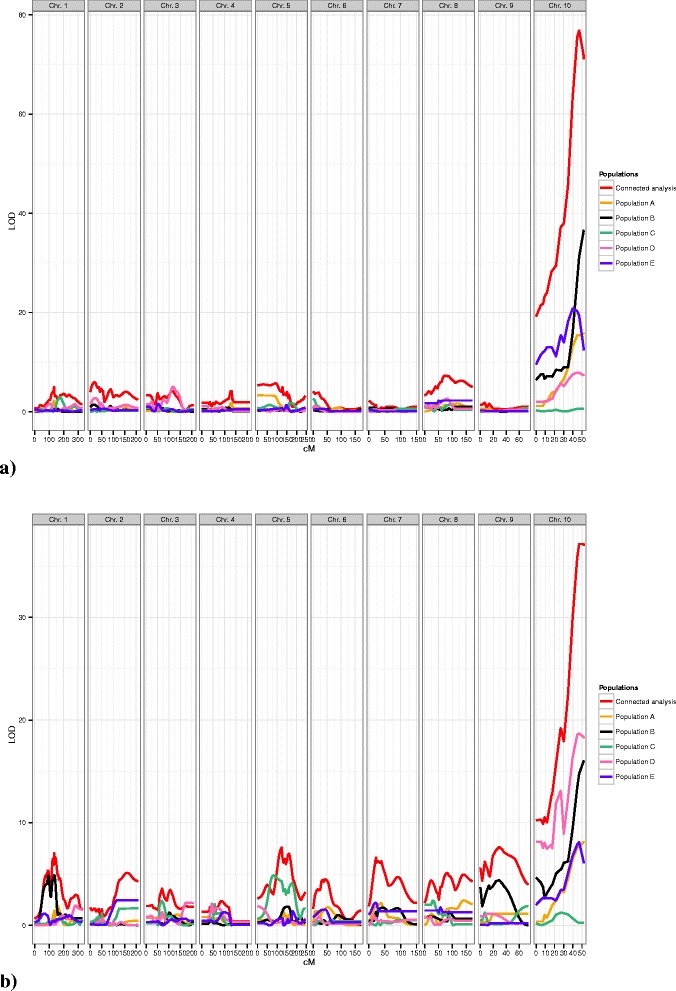
**Plot of the LOD scores of the trait extinction rate (EX) across the 10 chromosomes in the (a) field experiment compared to the (b) greenhouse experiment.** Each colored line represents a single population analysis, the red line represents the connected analysis.

In the connected analysis of the traits EX and IR, we observed a huge peak at the end of chromosome 10 in the plot of the LOD scores (Figure 4, Additional file 1: Figure S1 and Figure S3) in both, the field and greenhouse experiments. This QTL with significant additive effects whose CI comprised the genome region between the markers *PUT-163a-60352819-2700* and *SYN15407*, explained in the connected analysis about 45% of the phenotypic variance. The identification of this single QTL with significant additive effects for EX and IR suggests that BYDV resistance is oligogenically inherited.

The QTL CI with significant additive effects on chromosome 10 overlapped with the CI of all individual populations, except population C and explained between 23% and 68% of the phenotypic variance (Table 5). This suggests that this QTL interval is involved in the inheritance of BYDV in all these populations and that this genome region could be validated in different genetic material. Population C was the only population in which the QTL on chromosome 10 could not be detected. This finding indicates that the parental inbred Ky226 and FAP1360A carry the identical allele at the highly linked marker *SYN4811* and therefore no QTL can be detected in this cross.

These findings are in accordance with a previous GWAS by Horn et al., 2014 [15], where three SNPs in the same region on chromosome 10 explained 25% of the phenotypic variance for EX. This validation of the mentioned genome region on chromosome 10 in an independent group of genetic material leads to the assumption, that this major QTL for BYDV resistance in maize contributes to the resistance in various germplasm and might be broadly applicable in MAS projects of commercial breeding programs.

The QTL CI on chromosome 10 for EX and IR is located within the QTL CI for resistance to MDMV, identified by Zambrano et al., 2014 [11]. Furthermore, the locus *wsm3* for WSMV resistance [12,13] is located within the EX QTL on chromosome 10. The locus *mcd2* for MCDV resistance [14] and also three minor resistance QTL for SCMV [9,10] are reported to be associated with this genome region. This finding might suggest that the major QTL for BYDV on chromosome 10 possibly also leads to resistance of several other virus diseases. This, however, requires further research.

The QTL with significant additive effects for EX and IR on chromosome 10 colocalized with the QTL for the trait RE (Table 3, Additional file 1: Figure S2) which explained 30% of the phenotypic variance. Moreover, there was another QTL with significant additive effects on chromosome 2, explaining 11% of the phenotypic variance of RE. In the single population analysis, we identified these QTL with significant additive effects for RE on chromosome 2 and 10 only in population D. The reason could be that population D was the only population with the parental inbred W64A, which showed strong BYDV symptoms (RE).

In the greenhouse experiment, we identified a QTL with significant additive and dominance effects on chromosome 9 for IR which was not detected in the analyses of the data from the field experiments. Nevertheless, we could validate the QTL with significant additive effects from the field analysis on chromosome 10 for EX and IR in the greenhouse experiment in the connected analysis. And furthermore, in all single populations, except in population C, the QTL CI on chromosome 10 overlapped with the CI of the connected analysis. This confirms our findings from the field experiment under greenhouse conditions. In the single population analysis of the greenhouse experiment, a QTL with significant additive and dominance effects was identified on chromosome 1 in population B for EX. In the same region, Zambrano et al., 2014 [11] identified a QTL for MMV resistance. In this region a benzoxazionoid QTL in maize was identified [32] which causes aphid resistance and is associated with low levels of 2,4-dihydroxy-7-methoxy-1,4-benzoxazin-3-one glucoside methyltransferase leading to an increased aphid resistance by promoting callose deposition. This finding suggests that the QTL on chromosome 1 is possibly associated with aphid resistance but further research is needed to proof this hypothesis. The reason for the additional QTL in the greenhouse experiment could be the genotype-environment interaction which can lead to the detection of different QTL in the greenhouse compared to the field (Figure 4) [33].

A QTL with significant additive effects for EX was detected on chromosome 5 by the single population analysis of population C in which the major gene did not segregate. For this QTL on chromosome 5 at the position 60.8 cM (G/A), we detected a significant (*α*=0.01) epistatic interaction with the position at 80.7 cM on chromosome 6 (T/C) explaining 21% of the phenotypic variance (Table 6). We observed that all genotypes of population C which are homozygous (GG) at the QTL position on chromosome 5 and homozygous (TT) at the position on chromosome 6 showed a low (0.44) EX value. These genotypes have the same allele combination like the resistant parental inbred Ky226. In contrast, it can be observed that all genotypes which are homozygous at both positions AA/CC as the parental inbred FAP1360A showed high EX values.

## Conclusions

A genome region on chromosome 10 was identified in a linkage mapping approach with five connected segregating populations explaining 45% of the phenotypic variance for BYDV resistance traits. This region could also be confirmed in single population analyses and under greenhouse conditions. This study shows that BYDV resistance is oligogenically inherited and influenced by one major QTL. Therefore, the BYDV resistance trait EX is the ideal candidate to apply for marker assisted selection as BYDV is phenotypically difficult to assess but genetically rather simply inherited.

## Additional file

Additional file 1
**Supplementary figures.** **Figure S1.** QTL for the trait infection rate (IR) across the genome in the field experiment. **Figure S2.** QTL for the trait red edges (RE) across the genome in the field experiment. **Figure S3.** QTL for the trait infection rate (IR) across the genome in the greenhouse experiment.

## References

[1] Huth W (1994). Maisvirosen - Tendenz zunehmend. Pflanzenschutz-Praxis.

[2] Harrington R, Clark SJ, Welham SJ, Verrier PJ, Denholm CH, Hullé M (2007). Environmental change and the phenology of European aphids. Glob Change Biol.

[3] Beuve M, Naibo B, Foulgocq L, Lapierre H (1999). Irrigated hybrid maize crop yield losses due to barley yellow dwarf virus-PAV luteovirus. Crop Sci.

[4] Loi N, Osler R, Lapierre H, Lapierre H, Signoret PA (2004). Barley yellow dwarf associated to, BYDV-PAV virus. Virus and virus diseases of Poaceae (Gramineae).

[5] Panayotou PC (1977). Effect of barley yellow dwarf on several varieties of maize. Plant Dis Rep.

[6] Horn F, Habekuß A, Stich B (2013). Natural variation for BYDV resistance in maize. Maydica.

[7] Grüntzig M, Fuchs E, Werner M (1997). Occurence and influence of barley yellow dwarf luteovirus (BYDV) on growth and yield of maize. Nachrichtenblatt des deutschen Pflanzenschutzdienstes.

[8] Osler R, Loi N, Lorenzoni C, Snidaro M, Refatti E (1985). Barley yellow dwarf virus infections in maize (Zea mays L.) inbreds and hybrids in northern Italy. Maydica.

[9] Xia X, Melchinger AE, Kuntze L, Lübberstedt T (1999). Quantitative trait loci mapping of resistance to sugarcane mosaic virus in maize. Phytopathology.

[10] Zhang SH, Li XH, Wang ZH, George ML, Jeffers D, Wang FG (2003). QTL mapping for resistence to SCMV in chinese maize germplasm. Maydica.

[11] Zambrano JL, Jones MW, Brenner E, Francis DM, Tomas A, Redinbaugh MG (2014). Genetic analysis of resistance to six virus diseases in a multiple virus-resistant maize inbred line. Theor Appl Genet.

[12] McMullen MD, Simcox KD (1995). Genomic organization of disease and insect resistance genes in maize. Mol Plant Microbe In.

[13] Jones MW, Boyd EC, Redinbaugh MG (2011). Responses of maize (Zea mays L.) near isogenic lines carrying Wsm1, Wsm2, and Wsm3 to three viruses in the Potyviridae. Theor Appl Genet.

[14] Jones MW, Redinbaugh MG, Anderson RJ, Louie R (2004). Identification of quantitative trait loci controlling resistance to maize chlorotic dwarf virus. Theor Appl Genet.

[15] Horn F, Habekuß A, Stich B (2014). Genes involved in barley yellow dwarf virus resistance of maize. Theor Appl Genet.

[16] Flint-Garcia SA, Thuillet AC, Yu J, Pressoir G, Romero SM, Mitchell SE (2005). Maize association population: a high-resolution platform for quantitative trait locus dissection. Plant J.

[17] Stich B (2009). Comparison of mating designs for establishing nested association mapping populations in maize and Arabidopsis thaliana. Genetics.

[18] Stich B, Melchinger AE, Heckenberger M, Möhring J, Schechert A, Piepho HP (2008). Association mapping in multiple segregating populations of sugar beet (Beta vulgaris L.). Theor Appl Genet.

[19] Bardol N, Ventelon M, Mangin B, Jasson S, Loywick V, Couton F (2013). Combined linkage and linkage disequilibrium QTL mapping in multiple families of maize (Zea mays L.) line crosses highlights complementarities between models based on parental haplotype and single locus polymorphism. Theor Appl Genet.

[20] Blanc G, Charcosset A, Mangin B, Gallais A, Moreau L (2006). Connected populations for detecting quantitative trait loci and testing for epistasis: an application in maize. Theor Appl Genet.

[21] Gilmour AR, Gogel BJ, Cullis BR, Thompson R. ASReml User Guide Release 2.0. VSN International Ltd: Hemel Hempstead, HP1 1ES. UK; 2006.

[22] R Core Development (2011). Team R: a language and environment for statistical computing.

[23] Ganal MW, Durstewitz G, Polley A, Bérard A, Buckler ES, Charcosset A (2011). A large maize (Zea mays L.) SNP genotyping array: development and germplasm genotyping, and genetic mapping to compare with the B73 reference genome. PLoS One.

[24] Benke A, Urbany C, Marsian J, Shi R, Wirén NV, Stich B (2014). The genetic basis of natural variation for iron homeostasis in the maize IBM population. BMC Plant Biol.

[25] Givry SD, Bouchez M, Chabrier P, Milan D, Schiex T (2005). CarthaGene: multipopulation integrated genetic and radiation hybrid mapping. Bioinformatics.

[26] Jourjon MF, Jasson S, Marcel J (2005). MCQTL: multi-allelic QTL mapping in multi-cross design. Bioinformatics.

[27] Charcosset A, Mangin B, Moreau L, Combes L, Jourjon MF (2000). Heterosis in maize investigated using connected RIL populations. Quantitative genetics and breeding methods: the way ahead.

[28] Mangin B, Cathelin R, Delannoy D, Escalière B, Lambert S, Marcel J, et al. MCQTL: A reference manual. 2010. carlit.toulouse.inra.fr/MCQTL/.

[29] Smalley M, Daub J, Hallauer A (2004). Estimation of heritability in maize by parent-offspring regression. Maydica.

[30] Grüntzig M, Fuchs E (2000). Occurence of luteoviruses of cereals in Zea mays L. J Plant Dis Protect.

[31] Payseur BA, Nachman MW (2000). Microsatellite variation and recombination rate in the human genome. Genetics.

[32] Meihls LN, Handrick V, Glauser G, Barbier H, Kaur H, Haribal MM (2013). Natural variation in maize aphid resistance is associated with 2,4-dihydroxy-7-methoxy-1,4-benzoxazin-3-one glucoside methyltransferase activity. Plant Cell.

[33] Brachi B, Faure N, Horton M, Flahauw E, Vazquez A, Nordborg M (2010). Linkage and association mapping of Arabidopsis thaliana flowering time in nature. PLoS Genet.

