# Supplementary material for: Linkage mapping of Barley yellow dwarf virus resistance in connected populations of maize
Source: BMC Plant Biol. 2015 Feb 3;15:29. doi: 10.1186/s12870-015-0420-x (PMC4329211; doi:10.1186/s12870-015-0420-x)
Supplement: Additional file 1 — Supplementary figures. Figure S1. QTL for the trait infection rate (IR) across the genome in the field experiment. Figure S2. QTL for the trait red edges (RE) across the genome in the field experiment. Figure S3. QTL for the trait infection rate (IR) across the genome in the greenhouse experiment. [file 12870_2015_420_MOESM1_ESM.pdf]

Linkage mapping of *Barley yellow dwarf virus* resistance in connected populations of maize

Frederike Horn[1], Antje Habekuß[2] and Benjamin Stich[1*]

[1]Max Planck Institute for Plant Breeding Research, Carl-von-Linné-Weg 10, 50829 Cologne, Germany

[2]Julius Kuehn-Institute, Erwin-Baur-Str. 27, 06484 Quedlinburg, Germany

BMC Plant Biology

ADDITIONAL FILES

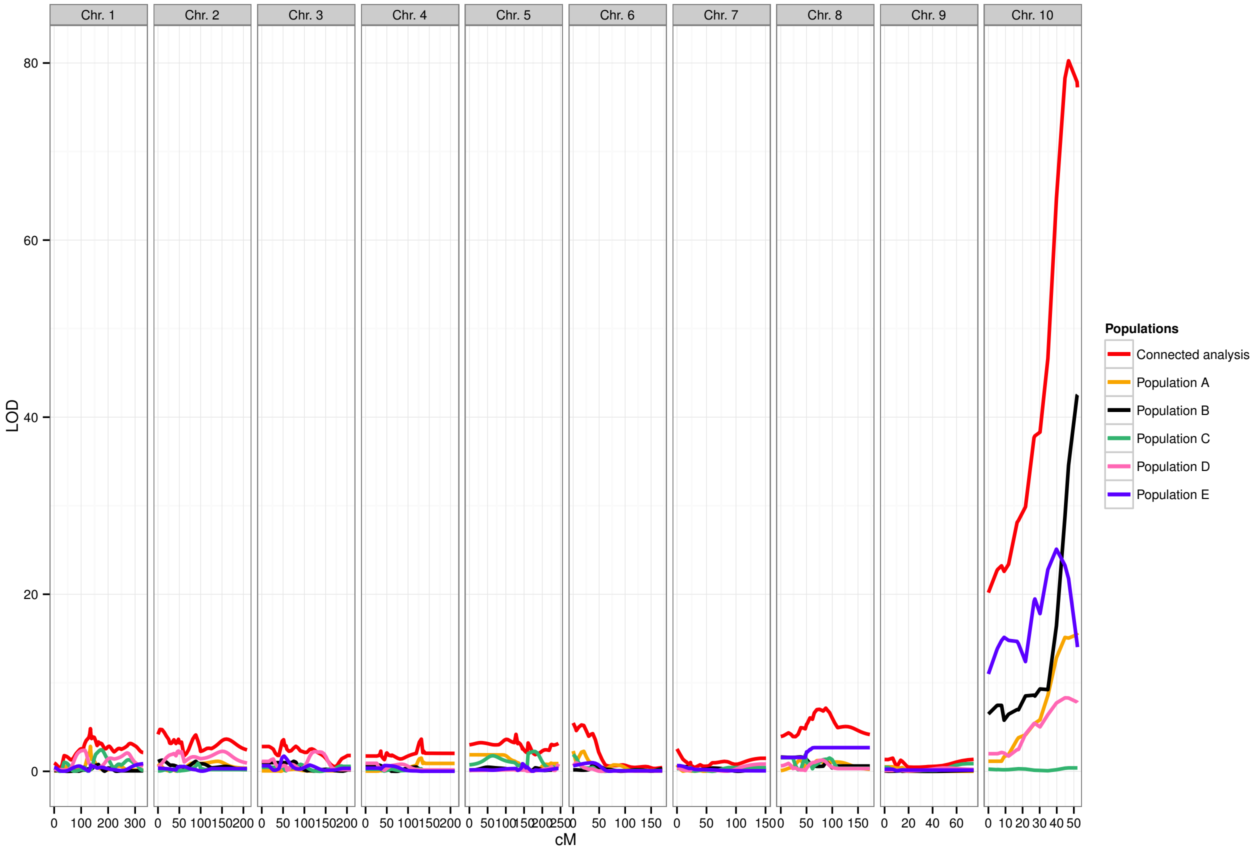

Figure S1 QTL for the trait infection rate (IR) across the genome in the inoculated field experiment.

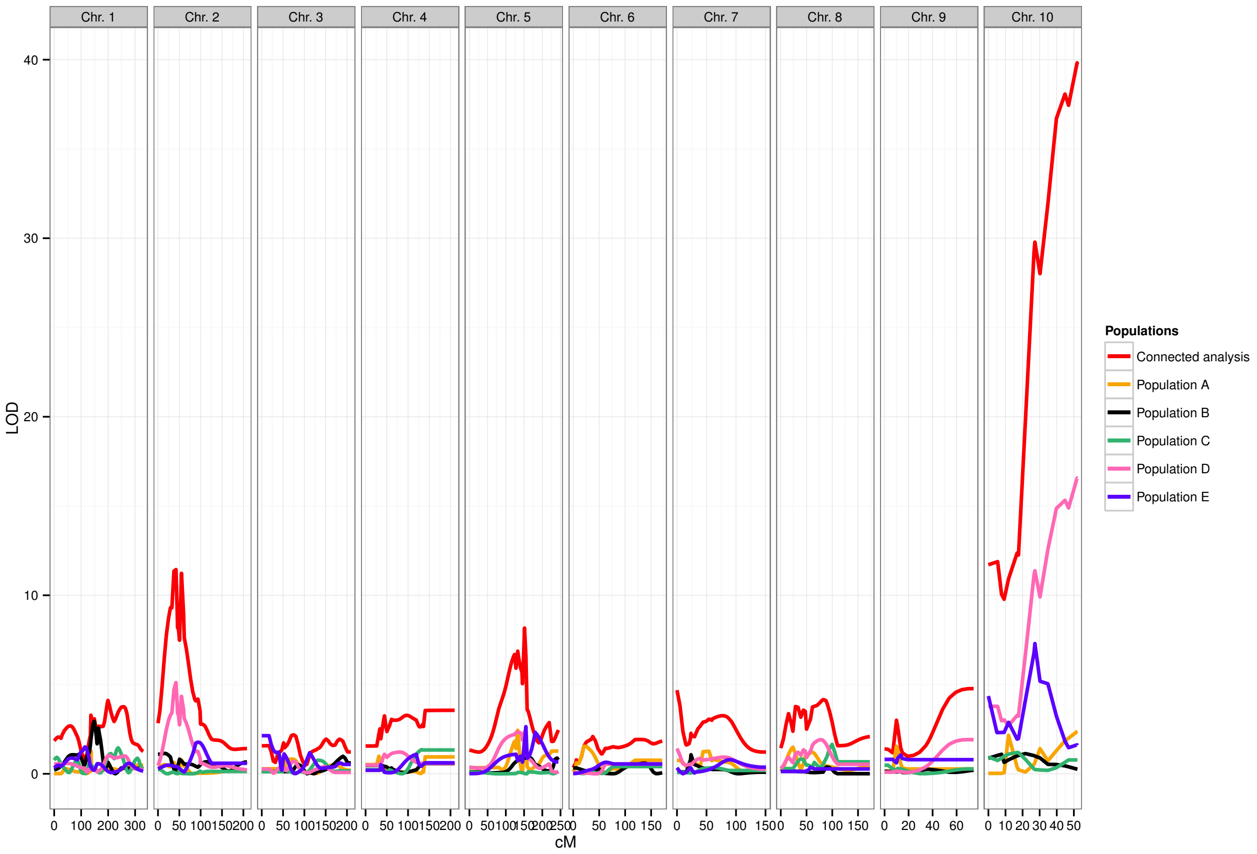

Figure S2 QTL for the trait red edges (RE) across the genome in the inoculated field experiment.

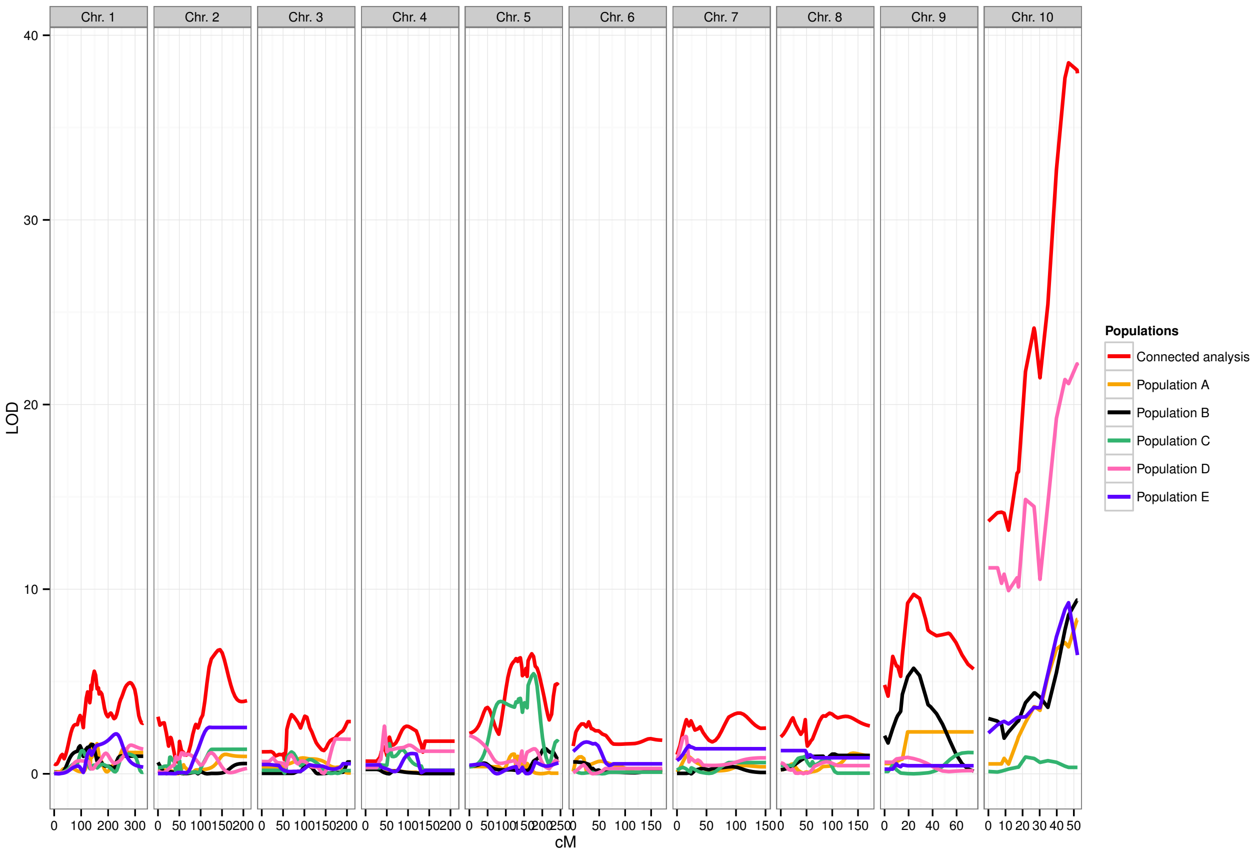

Figure S3 QTL for the trait infection rate (IR) across the genome in the greenhouse experiment.
